# Urban settlements’ vulnerability to flood risks in African cities: A conceptual framework

**DOI:** 10.4102/jamba.v9i1.370

**Published:** 2017-02-27

**Authors:** Rafiu O. Salami, Jason K. von Meding, Helen Giggins

**Affiliations:** 1 School of Architecture and Built Environment, University of Newcastle, Australia

## Abstract

In the recent past, the frequency and gravity of large-scale flood disasters have increased globally, resulting in casualties, destruction of property and huge economic loss. The destructive flood disaster devastating Louisiana, USA, is a recent example. Despite the availability of advanced technological capabilities for dealing with floods in developed nations, flood disasters continue to become more rampant and disastrous. Developing countries in Africa such as Benin, Ghana, Nigeria, Senegal and Sudan have recently experienced severe flooding, leaving a considerable number of human casualties and thousands displaced. In African cities, most vulnerable urban residents usually have lesser capacity and fewer resources to recover from the shocks of disaster as a result of the failure of governments to build human security for poor African residents. Many scholars have acknowledged the lack of appropriate vulnerability assessment frameworks and policies, questioning the efficiency and effectiveness of the tested models in Africa. The ability to accurately identify, measure and evaluate the various vulnerabilities of affected people and communities is a right step towards reducing disaster risk. This article aimed at developing a framework for assessing urban settlements’ vulnerability to flood risks in Africa. The framework is currently being tested to assess various dimensions of vulnerability drivers in three urban communities in Ibadan metropolis, the third largest city in Nigeria, focusing more on flood risk perceptions and behaviour of the risk bearers. It uses participatory and mixed method approaches to socially construct vulnerability of populations at risk. This model emanates from the evaluation of considerable relevant literature and an array of vulnerability assessment frameworks. It integrates some approaches that are applicable to African cities in a bid to create a versatile tool to assess, identify and mitigate the effects of flood disaster risk and reduce urban poor’s vulnerability to natural and human-induced hazards.

## Introduction

There is an increase in the magnitude and scale of natural and human-induced disasters, in particular the hydro-meteorological-related disasters such as floods and windstorms (Cutter, Boruff & Shirley 2003; Vos et al. 2010). It is widely acknowledged that floods are the most frequent and widespread disaster in the world, causing devastating effects on the lives of millions of people and their properties, as well as infrastructure and the natural environment (EM-DAT 2015; Vojinović 2015). The increase in population growth, rapid urbanisation, the spread of unplanned land use and consequent effects of change in climate are leading causes of natural and human-made disasters. The Intergovernmental Panel on Climate Change (IPCC) has warned on many occasions that the frequency and gravity of extreme weather events such as drought and excessive rainfalls resulting in flood and landslides are unstoppable because of the human interference with the climate system (Hardoy, Mitlin & Satterthwaite 2013; IPCC 2013; Mitlin & Satterthwaite 2013).

The ensuing risks of climate change and natural hazards like floods largely affect the urban poor living in cities particularly in developing countries because of their usual location in urban areas with unique spatial characteristics denoting informal settlements (Baker 2012). According to UN-Habitat (2011), informal settlements are residential houses where inhabitants lack basic services, security of tenure and non-compliance with building regulations. Most of these informal settlements inhabitants are vulnerable to multiple hazards because of their living conditions that are characterised by inadequate basic services, infrastructure and closeness to dangerous zones such as floodplains, rivers and other unsafe areas (Baker 2012).

For example, in some urban settlements in Ibadan metropolis, there is an array of congested poor houses which are unfit for habitation, characterised by unhealthy neighbourhood conditions, indiscriminate dumping of wastes and inadequate infrastructural facilities. More importantly, flood disaster is not a recent experience in Ibadan metropolis. According to many researchers, in Ibadan alone, more than 16 devastating flood disasters of varying degrees have occurred with records of more than 35 000 deaths and economic loss worth several millions of naira (Agbola et al. 2012; Ajayi et al. 2012; Eguaroje et al. 2015; Tomori 2008). Therefore, the urban settlements’ exposure to disaster risks is likely to intensify urban poverty, lack of societal resilience and their vulnerability.

In the recent times, the hydrological and meteorological disasters such as floods, droughts and weather storms have been prominent worldwide. For instance, in sub-Saharan Africa, the cumulative effect of the last decades indicates that floods and droughts alone are responsible for around 80% of disaster-related deaths and 70% of economic losses (Ndaruzaniye et al. 2010). The studies that detailed the better comprehension of the intensity and scale of urban settlements’ exposure to flood risks in African cities are still limited (Adelekan 2011; Nkwunonwo, Malcolm & Brian 2015). To achieve a detailed, sustainable and community participatory flood risk management, a better understanding of flood hazards, flood vulnerability and flood risk perception is essential. The objective of this article is to develop a framework that addresses the human settlements’ vulnerability to flood disaster risk in African cities by providing deep understanding of the concept; identification and assessment of the flood risks; exposure, susceptibility and adaptive coping capacity in the context of households’ or communities’ social, economic, cultural, institutional and physical vulnerabilities.

## Flood risks in African cities

Globally, floods account for more than 55% of all fatalities with nearly 2.5 billion people affected (EM-DAT 2015) and more than 30% of global economic losses from natural disasters (Hallegatte et al. 2013). Floods are the most frequent and widespread disaster in Africa, particularly in sub-Saharan Africa (Douglas et al. 2008). For example, an average of 500 000 people per year are affected by floods in West Africa alone (Jacobsen, Webster & Vairavamoorthy 2012). Meanwhile, the projected average annual population to be affected by river floods is around 21 million people worldwide and is likely to rise to 54 million by 2030 (World Resources Institute [WRI] 2016).

In sub-Saharan Africa alone, 654 floods have affected 38 million people with around 13 000 deaths recorded in the last 33 years (Tiepolo 2014). Tiepolo affirms that these outrageous figures necessitate the urgent need to seek for an effective solution to mitigate flood risk in the context of adaptation to climate change (Tiepolo 2014). In Nigeria, the flood disasters that occurred in 2012 affected 32 of the country’s 36 states, with 24 states severely affected, and an estimated total of 7.7 million people (Nkwunonwo, Whitworth & Baily 2015). In East Africa, according to Douglas et al. (2008), flooding and mudslides wreaked havoc in countries like Kenya, Burundi, Rwanda, Tanzania and Uganda, leaving tens of thousands of people displaced from their homes with more than 112 human casualties.

Flood risks in African cities have been largely exacerbated as a result of anthropogenic influence which immensely contributed to the flood disaster risk (Agbola et al. 2012; EM-DAT 2015). For example, human activities such as rapid urbanisation, uncontrolled urban growth, unregulated informal settlements on the low-lying floodplain areas, disregard to waste management and poor maintenance of drainage are major contributors to flood risk (Douglas et al. 2008; Eguaroje et al. 2015).

Urban settlements in African cities are commonly ravaged by flash, pluvial, fluvial and coastal flooding (Douglas et al. 2008). According to Few (2003) and Vojinović (2015), fluvial floods are also known as riverine flooding which is triggered by excessive rainfall over a couple of hours causing a river to exceed its limit, overtopping natural or artificial defences and inundating urban areas. Coastal floods usually affect cities that have close proximity to the ocean or the coastal environment as a result of storm surges influenced by the seasonal interruption (Vojinović 2015). Flash floods resulting from the direct rapid response to the high intensity of rainfall mostly occur in steep slopes. Pluvial floods usually occur in urban areas during intense rainfall which could overwhelm the capacity of drainage systems (Begum, Stive & Hall 2007; Houston et al. 2011; Merz, Thieken & Gocht 2007; Vojinović 2015). For instance, Ogunpa flood disaster that occurred in Ibadan, which claimed more than 200 human lives and destroyed assets worth millions of naira, was facilitated by combinations of flash, fluvial and pluvial flooding (Etuonovbe 2011).

## Concepts of vulnerability

A clear understanding of vulnerability is an important ingredient for a successful framework development in the context of assessing urban settlements’ vulnerability in African cities. The word ‘vulnerability’ has multi-dimensional definitions (Birkmann 2006b; Vogel & O’Brien 2004), and there is no single absolute explanation that is regarded as the best conceptualisation of vulnerability (Kasperson & Archer 2005). Many scholars have given an array of definitions for vulnerability in different context, for instance, the definition of vulnerability to natural and human-induced hazards in relation to climate change (IPCC 2001), in the context of environmental hazards (United Nations International Strategy for Disaster Reduction 2004) and with regard to floods (Connor & Hiroki 2005; Van der Veen & Logtmeijer 2005).

The vulnerability is commonly applied to a social system as a series of conditions and processes occurring from physical, social, economic and environmental circumstances, which increase the susceptibility of a society, property or environment to the impact of hazards (United Nations International Strategy for Disaster Reduction 2004; Wilson 2012). However, most climate researchers embrace a popular vulnerability definition given by the IPCC, which describes vulnerability as the degree to which a system is susceptible to, or unable to cope with, adverse effects of climate change (IPCC 2001). In a nutshell, considerable authors view vulnerability in the context of variation in exposure to hazards, while others see it as variation in humans’ capacity to cope with hazards (Few 2003).

A society or city is said to be vulnerable when its characteristics and circumstances make it susceptible to the damaging effects of a risk (Kidokoro 2008). Therefore, vulnerability in this study depicts circumstances triggered by various phenomena in the form of physical, social, economic, cultural and environmental factors which make a society, system or asset susceptible to natural and human-made hazards. With regard to flood risk, Parker (2000) affirms the significance of applying the environmental approach (social and physical environments) to determine the flood vulnerability of a household or community and that social aspect should be more explored in detail.

Several studies (Brooks 2003; Downing et al. 2005; Füssel 2007; Luers et al. 2003; Metzger, Leemans & Schröter 2005) have seen vulnerability as closely inclined to a set of conditions before it can successfully be expressed, assessed and analysed. Füssel (2007) posits that four dimensions are essential to explain a vulnerable situation. Firstly, system or unit of analysis such as a geographical area and women group; secondly, an attribute of concern such as housing quality, health issues and human livelihood; thirdly, the hazard of concern such as floods; and finally, the temporal reference such as a short period of time or long period of time of fluvial floods assessment. A good example of detailed nomenclature of a flood vulnerability can be adequately explained by emphasising a system’s vulnerability to a hazard (flood) in the context of a particular system (urban settlement), in a specific location (Ibadan), in a scenario of stressors (social or environmental) and in a period of time (August 2011) (Metzger et al. 2005).

The combination of quantitative and qualitative methods for measuring vulnerability is crucial, particularly when identifying and measuring risks and vulnerabilities before and after disasters have occurred (Birkmann 2007). Many researchers have tested this approach for better understanding the levels of vulnerability of population groups or communities and the specific climatic threat they encounter (Adger et al. 2004; Mustafa et al. 2011). Mixed methods approach considers the social aspects of the individuals, households or community and involves the participation of population groups expressing their perceptions to the risks within a specific region (Wisner & Birkmann 2006). More importantly, mixed methods, according to Creswell (2014), combine both quantitative and qualitative approaches to benefit from their strengths which will result in the emergence of multiple forms of vulnerability measurement. These include a deductive approach that uses indicators and inductive or participatory approach that involves vulnerable population identifying their own perspective of vulnerability and resilience (Kuhlicke et al. 2011).

Meanwhile, a community-based and participatory approach which combines quantitative and qualitative methods has been acknowledged as the best alternative for flood risk assessment (Vojinović et al. 2014). This approach facilitates a holistic analysis of flood vulnerability assessments to be achieved through integration of qualitative methods (qualitative expressions, perceptions, opinions, beliefs and feelings) for social vulnerability assessment and use of quantitative methods (e.g. questionnaire) to measure physical vulnerability aspects of households or the community at risk (Rufat et al. 2015; Vojinović 2015). According to Niyibizi, Mpeirwe and Ajambo (2013), to facilitate the implementation of disaster risk reduction project, an integrated and multi-disciplinary approach towards vulnerability assessment and adaptation planning needs to be considered. There are limited empirical studies that use participatory approaches and integration of mixed methods for flood vulnerability assessment, particularly for flood risk perception (Herslund et al. 2015; Rufat et al. 2015).

## Overview of the key vulnerability frameworks

In the last decades, several vulnerability frameworks have been developed to assess people’s susceptibility to multiple hazards. Despite the emergence of a considerable number of models to measure vulnerability, Birkmann (2006a) posits that researchers were still unable to describe the term accurately. This is evident as lack of availability of detailed vulnerability assessment tools at local levels (individual, household, and community) is prevalent (Ciurean, Schröter & Glade 2013). Most residents in cities of African countries are vulnerable to multiple hazards such as floods and droughts because of their varying conditions determined by biophysical and socioeconomic characteristics. The authors review some of the relevant vulnerability frameworks and integrate them to develop a new suitable model that will be useful to create effective and efficient flood risk management tools for African cities. The carefully selected frameworks include the Pressure and Release (PAR), the Borgadi, Birkmann and Cardona (BBC) and CLimate change and Urban Vulnerability in Africa (CLUVA) frameworks.

The PAR model consolidates on the empirical findings of Chambers (1989) who concludes that human settlements’ exposure to particular biophysical and social risk can be resisted based on individuals’ or households’ or community capacity to mitigate with various adaptive mechanisms (Chambers 1989). PAR model uses pseudo-equation (*R = H × V*) to define risk as a product of hazard and vulnerability (Blaikie et al. 1994, 2014). The PAR model views disaster as the intersection of opposing forces: a production of social processes on the one hand, and natural hazard event on the other. It further establishes three levels of progressions: root causes; dynamic pressures; and unsafe condition to explain the human vulnerability determinant factors and situations that increase disaster risks (Wisner et al. 2004). It is generally known as vulnerability conceptual framework that centres on explaining the determinant drivers of vulnerability. However, PAR model is just a tool for vulnerability explanation that lacks measuring capability. It also gives more weight to global and regional levels in terms of vulnerable analyses.

The BBC framework adopts the three cardinals of sustainable development (social, economic and environment aspects) linked with disaster reduction in its vulnerability assessment framework emphasising the environmental part of vulnerability (Green, Parker & Tunstall 2000). It further analyses the vulnerability concept that defined exposed system and people’s coping capacities, as well as differentiating between the period for risk preparedness (*t* = 0) and disaster or emergency management (*t* = 1). This signifies that vulnerability assessment is not just about damage evaluation (Ardestani, Fisher & Balzter). Despite the integration of many frameworks to create the BBC model, it does not indicate the association between livelihood and vulnerability. It lacks other vital vulnerability determinant factors that relate to institutional or political issues.

The CLUVA model was developed specifically for vulnerability assessment of urban systems, residents and assets in the context of natural and human-made disasters in Africa. It shares some similarities with the PAR and BBC frameworks in areas of vulnerability concept in terms of the exposure, susceptibility and coping/adaptive capacity, as well as assessment at three levels of population groups: individual, household and community. The CLUVA model identifies with four vulnerability dimensions to assess different levels of a unit of analysis (Jean-Baptiste, Kabisch & Kuhlicke 2013).

Given the global acknowledgement of the recurring and devastating effects of flooding and dire necessity to minimise vulnerability, there is a need to develop a robust flood vulnerability assessment that embraces socially constructed evaluation, participatory and mixed method approaches that enable effective comprehensive assessment of vulnerable population or area. Therefore, this study evaluates the applicability of the existing assessment framework, considering the emphasis on multi-dimensional nature (different dimensions of groups), scale dependence (unit of analysis) and dynamism of factors that influence vulnerability. The study integrates and develops the appropriate flood vulnerability assessment framework for African cities.

## Flood vulnerability assessment framework for African cities

**FIGURE 1:**
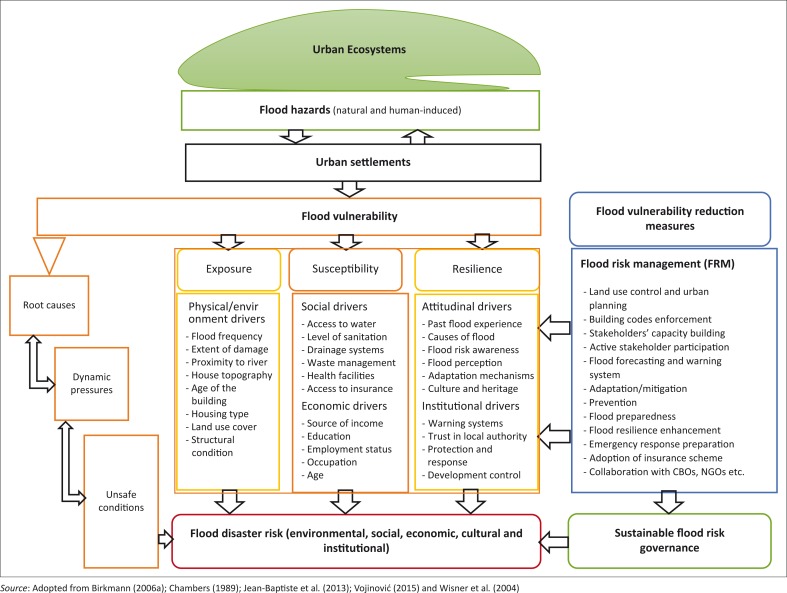
The flood vulnerability assessment: conceptual framework for African cities.

The aim of any vulnerability assessment is to identify why a population or a system is vulnerable to single or multiple hazards (Janssen & Ostrom 2006). The major purpose of developing this flood vulnerability assessment tool is to capture the real conditions of a specific population group in flooded area that is directly affected or likely to be affected by natural and human-induced hazards (hydro-meteorological hazards), so as to design disaster risk reduction strategies that can be applied in decision-making processes (Takemoto 2011). Given the fact that vulnerability is multi-dimensional and unequal, scale dependent and dynamic (Vogel & O’Brien 2004) and that application of the African context of flood vulnerability frameworks that embrace holistic approaches is still limited (UNISDR 2011), we therefore propose a flood vulnerability assessment framework (Figure 1) that emphasises a participatory and integrated approach for African cities. The framework is presently undergoing empirical tests to purposely explore different characteristics of residents’ vulnerability to flooding risk, starting with Ibadan metropolis, south-western Nigeria (targeting three urban communities). The objective of the framework includes flood risk identification; risk assessment; elements at risk identification; vulnerability assessment; comparative analysis of vulnerabilities between communities; and the creation of flood risk management tool.

This proposed flood vulnerability assessment framework exemplifies how urban settlements in typical African cities interact with natural and human-induced hazards which could cause disasters (such as urban floods) that are likely to affect vulnerable urban poor residents (Blaikie et al. 1994). The poor residents’ flood vulnerability is as a result of social processes and underlying causes which Birkmann (2006b) describes the three progressions of vulnerability: root causes, dynamic pressures and unsafe conditions. This framework adopts the three stages of vulnerability (Figure 1) as applies to urban settlements in African cities. For instance, underlying root causes of flood vulnerability in African cities are triggered by differential access to livelihood income, tenure security and bad governance, among others (Baker 2012). The progression of the flood vulnerability ranges from root causes to dynamic pressures (such as unhealthy and urban growths, demographic pressure) and unsafe conditions (such as hazardous locations and deficient housing and infrastructure), which could lead to varying scales of flood disaster risk. Flood disaster risk reduction interventions such as structural and non-structural can reduce flood vulnerability through the application of result-oriented flood risk management (FRM) tools.

This African context of flood framework assessment uses vulnerability as a precursor for risk reduction (Ciurean et al. 2013), and puts flood risk bearers (human system) on the central stage, focusing on adaptive coping capacity of the society and ability to resist, respond and recover from impact of natural hazards such flooding (Blaikie et al. 1994). It also recognises that flood vulnerability is determined by three factors: (1) the degree of exposure; the probability that population groups and their assets like properties, infrastructure and cultural heritage will be struck by flooding (Penning-Rowsell et al. 2005). The flood duration, intensity, velocity, frequency and water level of a flooded area are measurable and (2) susceptibility; relates to the extent to which economic values, building structures and people in a flood-prone region are likely to be harmed by flood hazards (Balica & Wright 2010; Begum et al. 2007). The population density, economic values and building structures in a specific flooded area can be measured and (3) resilience; relates to a long time social, economic, technological and cultural adaptive coping mechanisms to mitigate flood risk (Cardona 2001). The adaptive coping mechanisms are largely influenced by individuals’ or households’ flood risk perceptions, flood awareness, flood knowledge, experience and coping strategies to minimise flood risk. These flood risk perceptual indicators or criteria are difficult to measure through quantitative methods (Rufat et al. 2015).

Each of the three factors of flood vulnerability provides different vulnerability drivers with varieties of selected indicators. It is important to acknowledge that vulnerability is circumstance-specific; therefore, indicators or variables should be selected based on specific type of hazards such as flooding, and should also be based on specific issues such as flood risk preparedness or flood preparatory measures (Buckle, Mars & Smale 2000; King & MacGregor 2000; Oulahen 2015). The carefully selected indicators are categorised by the researcher under five vulnerability drivers (Figure 1). These are physical, social, economic, attitudinal and institutional drivers of vulnerability, which were adapted and integrated from an array of studies (Birkmann 2007; Chambers 1989; Jean-Baptiste et al. 2013; Vojinović 2015) to be appropriate for the community-based flood vulnerability assessment framework in the context of African cities.

The authors integrate attitudinal drivers to understand psycho-social behavioural indicators such as cultural beliefs, flood risk perceptions, awareness and adaptive coping mechanisms through participatory approaches to achieve more robust evaluation tools regarding social vulnerability. Also, institutional drivers are included as part of determinant factors of flood vulnerability in African cities because the production of the urban settlement itself is deeply political and, at the same time, it is almost invisible to residents to scrutinise (Heynen, Kaika & Swyngedouw 2006). However, the introduction of urban political ecology to this study provides a critical lens to understand African cities in the context of socio-spatial-political purposes and outcomes (Evans 2015). It provides a platform to carry out issues relating to social and environmental justice, as well as dealing with the complexity of urban habitat and systems such as flood vulnerability (Keil 2003). The indicators considered under institutional drivers include trust in local authority, development controls, risk mitigation strategies, income diversification et cetera.

Measuring vulnerability according to studies (Birkmann 2005; Kienberger & Steinbruch 2005) is categorised into two approaches: expert-based knowledge approach such as the use of indicators, and participatory approach where people at risk are involved in giving their own experience and perceptions. Our proposed framework incorporates combinations of both indicator-based expert and participatory approaches in order to capture the natural and socio-natural aspects of the human settlements’ vulnerability to flood risk. This study also recognises the diversity and representations of vulnerability profiles at varying scales (unit of analysis) ranging from individual, household, community to city, and at different levels (a type of component) such as social, economic or cultural (Balica & Wright 2010; Wisner et al. 2004).

### Flood vulnerability determinant drivers in African cities

Based on the reviews of a variety of studies, this study agrees with growing evidence on cities’ flood vulnerability which says most flood-related disasters are not mainly caused by natural disasters alone. The major determinant factors are largely attributed to human activities that involve social-eco-political, historical and cultural forms (Birkmann 2007; Milly et al. 2002; Seyoum et al. 2011; Vojinović 2015; Vojinović & Abbott 2012). While the lack of basic knowledge and understanding of flood risk by the people living in flood-prone areas may have contributed to the ineffective decisions, Pelling and Wisner (2012) posit that poor governance and social and environmental injustice are underlying root causes of the flood risk. For instance, a city with a very low quality of basic or infrastructure services, unplanned growth and rapid urbanisation coupled with effects of climate change can turn a heavy rainfall into a catastrophic flood (Baker 2012; Global Footprint Network 2012). Therefore, in order to have a deep understanding of the interactions between natural and social-related underlying causes of flood vulnerability and risk, this study takes both physical and social vulnerabilities as key determinant factors of flood risk. For clear identification of indicators or criteria and easy vulnerability assessment, these factors are further categorised into five dimensions of flood vulnerability drivers in African cities: physical or environmental, social, economic, institutional and attitudinal drivers.

### Identifying flood vulnerability indicators and criteria

The precondition to achieving disaster risk reduction is the ability to measure vulnerability effectively. It is also important to have a deep understanding on how an array of flood vulnerabilities can be carefully identified, selected and assessed (Pandey, Manandhar & Kazama 2014). The indicator-based flood vulnerability assessment has been acknowledged as the most appropriate for evaluating populations group at all levels (UNISDR 2005), and particularly serves as a policy-making tool to initiate public awareness, as well as assisting government to prioritise budget allocations (Nasiri & Shahmohammadi-Kalalagh 2013). However, measuring vulnerability approaches also involves qualitative means and other broader assessment techniques (Birkmann 2006a). For instance, the Hyogo Framework for Action 2005–2015 (HFA) report emphasises the need to use indicators to assess the impact of disaster risks with respect to social, economic and environmental aspects of people at risk (UNISDR 2005). Therefore, identification and careful selection of a suitable set of qualitative criteria are also important for an effective and successful vulnerability assessment (Moser 2011; Wisner & Birkmann 2006).

This framework adopts five components of vulnerability drivers through which different variables and indicators or criteria (Table 1) can be evaluated so as to understand the underlying root causes of flood vulnerability, as well as detailing the vulnerability profiles of urban settlements in African cities at varying scales and levels. Considerable authors have identified several indicators (quantitative) to assess various dimensions of vulnerability (Adger 2006; Birkmann & Vulnerability 2006; Cutter et al. 2003). In other studies (Chambers 1989; Moser 2011; Wisner 2006; Wisner & Birkmann 2006), they used criteria (qualitative) to gain a better understanding of the perceptions of flood-prone victims.

**TABLE 1:** Identification of flood vulnerability indicators or criteria.

Variables	Indicators or criteria	Sources
1. Physical or environment	Housing	Ayoola and Amole (2014)
	• Age of the building	Owoeye (2013)
	• Housing type	Adelekan (2010)
	• Construction materials	Birkmann (2006b)
	• Structural condition	Grosh and Glewwe (2000)
	• Roofing material	Govender et al. (2010)
	• Neighbourhood quality	-
	• Land use cover	-
	• Land ownership	Ologunorisa (2004)
	• Density	-
	• Building codes	Grosh and Glewwe (2000)
	• Road network and transport	Balica and Wright (2010)
	Flooding	Brouwer et al. (2007)
	• Elevation of settlement above sea level	Agbola et al. (2012)
	• Proximity to the river	Ouma and Tateishi (2014)
	• The frequency of flood occurrence	-
	• Intensity	-
	• The extent of the damage	-
2. Economic	Source of income	Owoeye (2013)
	Level of education	Brouwer et al. (2007)
	Occupation	Kellens et al. (2011)
	Employment status	Adelekan (2010)
	Demographic structure	Ologunorisa and Adeyemo (2005)
	• Age	Shabu and Tyonum (2013)
	• Gender	Marfai et al. (2008)
	• Household size	Ho et al. (2008)
	• Household composition	Grosh and Glewwe (2000)
	• Population	Govender et al. (2010)
	• Race	Birkmann (2006b)
	Community participation	-
	Local resource base	-
	Access to insurance	-
3. Social	Basic or infrastructure services	Agbola et al. (2012)
	• Access to water	Grosh and Glewwe (2000)
	• Source of water	Govender et al. (2010)
	• Waste management	Birkmann (2006a)
	• Level of sanitation	Wisner (2006), Jean-Baptiste et al. (2013)
	• Drainage system	Adger (2006)
	• Health facilities	-
	• School	-
	• Transportation	-
4. Attitudinal	Past flood experience	Balica and Wright (2010)
	Flood risk awareness	Adelekan (2010)
	Level of preparedness	Kellens et al. (2011)
	Flood perception	Marfai et al. (2008)
	Causes of flood	Ologunorisa and Adeyemo (2005)
	Adaptation mechanisms	Agbola et al. (2012)
	Culture and heritage	Ho et al. (2008)
	Social network	-
5. Institutional	Effectiveness	Kellens et al. (2011)
	Trust in local flood risk management	Agbola et al. (2012)
	Protection and response	Birkmann et al. (2006b)
	Warning system	Wisner (2006)
	Development control	Vojinović (2015)
	Risk governance	Pelling and Wisner (2012)
	Evacuation route	Jean-Baptiste et al. (2013)
	Collaboration with NGO, CBO et cetera	-
	Participatory decision-making	-

NGOs, Non-Governmental Organisations; CBOs, Community Based Organisations.

## Conclusion

Most cities and urban centres in Africa are regarded as flood disaster risk hotspots (Baker 2012) because of rapid urbanisation, human activities and their vulnerability to the impacts of multiple hazards (Adelekan et al. 2015; Pelling & Wisner 2012; Vojinović 2015). These cities are increasingly overstretched to cater for high-density populations with inadequate infrastructure and basic services. Given the high percentage of informal settlements with corresponding substandard houses, inadequate protection of assets and development of unhealthy urban growth in African cities, flood mortality may continue to rise. Meanwhile, most urban residents have less capacity and few resources to mitigate or recover from shocks. In order to reduce the flood disaster risk and increase resilience, there is a need to develop an effective flood vulnerability assessment framework for deeper understanding of dominant root causes of flood hazards.

In this study, the authors review, examine, integrate and build on the existing relevant vulnerability assessment models, and develop the appropriate flood vulnerability assessment framework for African cities. The unique introduction of cultural or attitudinal determinant factor of flood vulnerability will enhance a good comprehension of the socially constructed vulnerability of population at risk using participatory approaches. Also, the study contributes to existing frameworks by integrating urban political ecology to gain a deeper understanding of African cities in the context of socio-spatial-political profiles through assessment of institutional drivers of flood vulnerability. The study identifies relevant indicators for evaluation of flood vulnerability and also justifies the significance of using a combination of quantitative and qualitative approaches to capture the comprehensive understanding of coupled human and natural systems that are exposed to flood hazards (Birkmann 2007). The proposed flood vulnerability assessment framework for African cities is currently being tested empirically in three urban communities (Bere, Mokola and Bashorun) in Ibadan metropolis, the third largest city in Nigeria. This analytical framework also recognises the dire need to know the proximate and underlying root causes, as well as the determinant factors of flood vulnerability in all aspects ranging from natural, technical, social, economic, cultural and institutional drivers. The outcomes of the flood vulnerability assessment will hopefully lead to the creation of flood risk management tools that combine structural and non-structural measures so as to reduce flood vulnerability and minimise impacts of the risk.
